# Atlas of Urinary Sediments

**Published:** 1900-03

**Authors:** 


**Atlas of Urinary Sediments.** By Dr. Hermann Rieder. Translated by Frederick Craven Moore, M.D. Edited and Annotated by A. Sheridan Delépine, M.B. Pp. viii., 111. London: Charles Griffin & Company, Limited. 1899.

The author is assistant in the medical clinic of Prof. v. Ziemssen, and this atlas will be found most useful to all clinical teachers, students, and medical practitioners. It contains 36 plates, comprising 167 figures, which with other figures in the text practically represent all the sediments found in the urine. The plates are beautifully executed, very clear, and many of them printed in colours; they will be found invaluable for reference in investigating the nature of any particular deposit. The arrangement of the book facilitates such reference. The drawings have so far as possible been made under a uniform moderate magnification, such as most practitioners use in clinical work. Special attention has been paid to the polymorphic forms of the more common deposits. Following the plates comes a full description of the various deposits met with in urine, especially dealing with their characters, mode of occurrence and pathological significance. The editor, Dr. Sheridan Delépine, has contributed to the text numerous and valuable additions, which are enclosed in brackets, and has also added some figures and an alphabetical index. In all respects the book is a good and useful one, and the most complete on the subject. The publishers also must be congratulated on the general style of the work, and the way in which the plates have been executed. We feel sure that to look through the book is to desire to possess a copy of it.

